# Overlapping consensus in pluralist societies: simulating Rawlsian full reflective equilibrium

**DOI:** 10.1007/s11229-023-04415-9

**Published:** 2023-12-21

**Authors:** Richard Lohse

**Affiliations:** Department of Philosophy, Karlsruhe Institute of Technology, Karlsruhe, Germany

**Keywords:** John Rawls, Overlapping consensus, Justice as fairness, Reflective equilibrium, Computational philosophy, Bounded rationality

## Abstract

The fact of reasonable pluralism in liberal democracies threatens the stability of such societies. John Rawls proposed a solution to this problem: The different comprehensive moral doctrines endorsed by the citizens overlap on a shared political conception of justice, e.g. his justice as fairness. Optimally, accepting the political conception is for each citizen individually justified by the method of wide reflective equilibrium. If this holds, society is in full reflective equilibrium. Rawls does not in detail investigate the conditions under which a full reflective equilibrium is possible or likely. This paper outlines a new strategy for addressing this open question by using the formal model of reflective equilibrium recently developed by Beisbart et al. First, it is argued that a bounded rationality perspective is appropriate which requires certain changes in the model. Second, the paper rephrases the open question about Rawlsian full reflective equilibrium in terms of the model. The question is narrowed down by focusing on the inferential connections between comprehensive doctrines and political conception. Rawls himself makes a demanding assumption about which connections are necessary for a full reflective equilibrium. Third, the paper presents a simulation study design that is focused on simplicity. The results are discussed, they fit with Rawls’s assumption. However, because of the strong idealisations, they provide a useful benchmark rather than a final answer. The paper presents suggestions for more elaborate study designs.

## Introduction

In liberal democracies there is bound to be a pluralism of incompatible yet equally reasonable comprehensive moral doctrines: This fact of reasonable pluralism is a challenge to the stability of such societies. How can citizens nonetheless agree on basic matters of justice? This is the question that Rawls poses himself in his 1993*Political Liberalism*. His answer is: by overlapping on a shared political conception of justice. Such a conception is freestanding and can, in principle, be incorporated in any reasonable doctrine. Optimally, all citizens do in fact incorporate such a shared conception and are also individually justified in doing so. This means, according to Rawls’s view on justification, that they are in a state of wide and general, or full, reflective equilibrium. However, Rawls focuses mainly on formulating a freestanding conception of justice and leaves somewhat open under which conditions a full reflective equilibrium would form.

Recently, a formal model of the method of reflective equilibrium was developed by Beisbart et al. (2021). The present paper aims to make this model fruitful for researching the open question in Rawls’s *Political Liberalism*. In particular, the question is framed appropriately and I describe in detail how the model can be used to address it. A simulation study illustrates the strategy, gives first results and can serve as the starting point for further research.

The paper is structured as follows. First, in Sects. 2–4, I give a short upshot of Rawls’s view on justifying conceptions of justice. His solution to the challenge of reasonable pluralism, including the notions of overlapping consensus and full reflective equilibrium, is presented in some more detail. I describe which issue deserves further attention: Under which conditions are citizens in a society likely to be in full reflective equilibium? I point out that Rawls makes a demanding assumption about which inferential connections between comprehensive doctrines and political conception are necessary for a full reflective equilibrium. It is argued that this assumption should be treated as a hypothesis that needs to be tested. Second, in Sects. 5–8, I present the formal model of reflective equilibrium. It is argued that a bounded rationality perspective is appropriate which forces certain changes in the model. The open question is recast in terms of the model. Finally, in Sects. 9–11, I present the design of a first study that simulates RE processes. The design focuses on simplicity and addresses Rawls’s hypothesis. The results are presented and explained. I argue that they validate the general research strategy. Additionally, the results seem to verify the hypothesis. However, because of the strong idealisations, they provide a useful benchmark rather than a final answer. Suggestions for de-idealisations are made. Section 12 concludes.

## Justice as fairness and reflective equilibrium

In his 1971*A Theory of Justice*,^1^ John Rawls formulates and attempts to justify *justice as fairness* (JF), a theory of justice concerning the basic structure of society. The theory consists of two principles of justice, lexically ordered in priority. Often labelled a version of ‘egalitarian liberalism’, JF is an alternative to the utilitarian paradigm.

How is JF supposed to be justified? According to Rawls, the relevant method of justification in this context is the *method of reflective equilibrium* (MRE): one compares a theory to one’s considered judgments about the subject matter. If there is a misfit, either can be revised. By going back and forth between the two levels and adjusting one to the other, one eventually reaches a *state of reflective equilibrium* (RE) where both levels fit. A theory is justified to the extent that it is the result of such a process, or can be rationally reconstructed as such (cf. TJ 18).

Rawls proposes a certain expository device that is supposed to help in the equilibration process: the initial situation. This is a hypothetical scenario in which some agents collectively choose principles of justice. This choice problem can be described in various ways and contain various assumptions about the motivation, knowledge, and so forth, of the agents. Given some description, the agents will choose a utilitarian principle, given some other description, they will choose Rawls’s JF. Of course, Rawls is particularly concerned with spelling out the latter description, which he calls the *original position*, containing amongst others his famous veil of ignorance.

Given this picture, MRE will bring three pieces into equilibrium: the description of the initial situation, the principles that would be chosen in this situation, and our considered judgments. Using the expository device of the initial situation, we are going back and forth not between judgments and theory directly, but between judgments and the description of the initial situation, which in turn yields principles which in turn fit or do not fit our judgments. However, it is important to note that the description of the initial situation itself does little justificatory work on its own. Some description might be offhand more plausible than another, but Rawls is very clear that MRE is what ultimately justifies the principles (TJ 19).

## Reasonable pluralism and overlapping consensus

Fast-forward to his 1993 follow-up monograph: *Political Liberalism*.^2^ Here Rawls addresses what he considers fundamental shortcomings in TJ. The main challenge arises from the fact of *reasonable pluralism*. That is, in the kind of liberal democracies that Rawls himself considers just, the free exercise of human rationality leads to a diversity of incompatible yet equally reasonable comprehensive moral doctrines. According to Rawls, the theory he put forward in TJ is but one of these comprehensive doctrines (PL xvi, xvii).

One main problem with reasonable pluralism is the matter of social *stability*. For a society to be stably just, it is necessary that most of its citizens agree on a shared conception of justice. Moreover, it is desirable that this agreement is not a mere compromise between conflicting interests, because such a compromise depends on a power balance which may shift and the compromise be lost. Instead, citizens optimally accept the shared conception of justice, because it fits into their respective comprehensive doctrines and can be justified from within it (PL 147). That is, the shared conception should not only be compatible, but in reflective equilibrium with all comprehensive doctrines in a society. Rawls calls this a *consensus for the right reasons*.

How exactly is this supposed to work? The shared conception has to be what Rawls calls a *political* conception of justice. This means, first and foremost, that it is doctrine-neutral. In particular, it provides the content of *public reason*, the doctrine-neutral grounds for adjudicating questions of basic justice. Importantly, since a political conception is conceptually unbiased, it can fit as a module in different comprehensive doctrines. This way the comprehensive doctrines may overlap on the political conception of justice, forming an *overlapping consensus*. In addition to agreeing on the conception, citizens should be in RE about their views. If they carefully considered the proposed views and arguments of their fellow citizens, their RE is called wide. If they do in fact overlap on a shared political conception of justice, their RE is general. If RE is wide and general, it is called *full* (PL 384n). Thus, in an optimally stably just society, citizens are in full RE about the underlying conception of justice.

## Open question

In PL, Rawls focuses on reformulating JF as a political conception of justice. In particular, he tries to make it freestanding. However, he delimits his ambitions explicitly:

The other point of a reasonable overlapping consensus is that PL makes no attempt to prove, or to show, that such a consensus would eventually form around a reasonable political conception of justice. The most it does is to present a freestanding liberal political conception that does not oppose comprehensive doctrines on their own ground and does not preclude the possibility of an overlapping consensus for the right reasons. (PL xlv f.)

Rawls only shows that JF can be freestanding, i.e. it is not on conceptual grounds impossible for different doctrines to overlap on it as a shared module. Importantly, he does not argue in detail that an overlapping consensus is guaranteed or even likely to develop in liberal democracies. Therefore, the following is an open question:

*Under which conditions are citizens in a society likely to be in full RE?*

This is an important question. If it turned out that, for whatever reasons, a full RE is very unrealistic or even impossible, then the Rawlsian solution to the problem of reasonable pluralism fails. If, on the contrary, there are realistic conditions that make a full RE likely, then these might contribute to a guideline for stabilising pluralist societies.

Of course, it is a very broad question with many interesting aspects to be studied, some of which Rawls himself has worked or at least commented on. In the rest of this section, I will further delineate the subject matter of this paper: First, I clarify that two important Rawlsian concepts, that of *public reason* and that of *public political culture*, will not be in focus here. Second, I discuss Rawls’s assumption that comprehensive doctrines, in order to be part of an overlapping consensus on a political conception, need to support that conception, rather than being neutral about or incompatible with it. This hypothesis will motivate the study design in Sect. 9.

Let’s start with why public reason will not be in focus here. Public reason, according to Rawls, is a part of the political conception of justice, next to the substantive principles of justice he defended in TJ (PL 224f). It gives citizens (including, importantly, government officials) the resources to reason about questions of basic justice without relying on any particular comprehensive doctrine. In fact, the principles of justice themselves can be justified by public reason. However, this is not the kind of justification I am interested in. Rawls himself distinguishes three kinds of justifications of the political conception (PL 386f):

- *Pro tanto* justification: The political conception is justified using the resources of public reason alone, i.e. referencing only political values. In terms of MRE, public reason is in reflective equilibrium with the principles of justice. (Since principles and public reason make up the political conception, in a sense the conception itself is in RE.) The justification is *pro tanto* because, in principle, the political values may be overriden by non-political ones once the political conception is not considered in isolation, but in a wider view.
- Full justification: A political conception is fully justified by an individual citizen if it is embedded in their comprehensive doctrine. That is, the different parts of the comprehensive doctrine and the political conception are in reflective equilibrium. If all citizens have in this sense fully justified the political conception, then there is an overlapping consensus, or consensus for the right reasons. They are in full reflective equilibrium.
- Public justification: A political conception is publicly justified by a political society (as a collective, not individual citizens). This public justification, as Rawls imagines it, is based on there being an overlapping consensus and on the ideas of stability for the right reasons (see above) and legitimacy (PL 388f). The details need not concern us here, the important point is that public justification is both different from and dependent on the full justification of individual citizens in an overlapping consensus.

Note that both *pro tanto* justification and public justification belong to the *public sphere*, because they must not presuppose any particular comprehensive doctrine. Full justification, on the other hand, may do so and thus belongs to the *private sphere*. Also note that not only public justification depends on there being a society-wide full justification of, or overlapping consensus on, the political conception. *Pro tanto* justification, too, depends on this, because it uses the resources of public reason and public reason is a part of the political conception. If there is no overlapping consensus on the conception, a reference to public reason will not be convincing to all citizens and the *pro tanto* justification will not be public in the proper sense. Thus, society-wide full justification is a precondition for the other two kinds of justification.

Now, for the purpose of this paper I am interested in this precondition, in the possibility of an overlapping consensus. That is, I am interested in the possibility of all citizens having fully justified the same political conception even though they endorse a variety of comprehensive doctrines. In particular, the two justifications of the public sphere and the structure and content of public reason are not directly relevant here. Thus, public reason itself will not be modelled in Sect. 9. (Nonetheless, it is in a weak sense represented, because the political conception, of which it is a part, does appear as an entity in the model.) Of course, public reason may be *indirectly* relevant for an overlapping consensus. In particular, public reason may be relevant for the functioning of the *public political culture* in a society and this culture in turn may be relevant for the possibility of an overlapping consensus. So let’s turn to this concept.

The public political culture of a society comprises “the political institutions of a constitutional regime and the public traditions of their interpretation (including those of the judiciary), as well as historic texts and documents that are common knowledge” (PL 13f). One might think that growing up and living exposed to a public political culture will have an influence on the likelihood of accepting the political conception that is realised in this culture. In fact, when explaining how an overlapping consensus might come about (PL §§6–7), Rawls speculates that living in a public political culture might be a crucial driving force behind the formation of an overlapping consensus:

This suggests that many if not most citizens come to affirm the principles of justice incorporated into their constitution and political practice without seeing any particular connection, one way or the other, between those principles and their other views. It is possible for citizens first to appreciate the good those principles accomplish both for themselves and those they care for, as well as for society at large, and then to affirm them on this basis. Should an incompatibility later be recognized between the principles of justice and their wider doctrines, then they might very well adjust or revise these doctrines rather than reject those principles. (PL 160)

It seems that one needs to take the public political culture in a society into account when investigating the possibility of an overlapping consensus.

However, this conflates two fundamentally different aspects of belief: *genesis* (whether and how beliefs come into existence) and *justification* (whether and how beliefs are justified or in reflective equilibrium). Both aspects are important, because we would like the beliefs in an overlapping consensus to be both existent and justified. In the above quote, Rawls describes the role of public political culture in the genesis of an overlapping consensus. This paper, however, is concerned with justification and not genesis.

Thus, citizens in my model will run their RE processes *in abstracto* and not embedded in a political culture. Consider the following two interpretations of MRE:

- Actual MRE: Beliefs are justified iff they are the result of an equilibration process. MRE describes the actual process of generating justified beliefs.
- Hypothetical MRE: Beliefs are justified iff they could have been the result of an equilibration process. MRE is a test for whether beliefs are justified, no matter how they were generated.

I know of no philosopher who interprets MRE as the one and only method of actually generating justified beliefs. Rather, the claim is usually weaker and some version of Hypothetical MRE. For example, after describing MRE in TJ, Rawls writes:

I shall not, of course, actually work through this process. Still, we may think of the interpretation of the original position that I shall present as the result of such a hypothetical course of reflection. (TJ 18)

I, too, interpret MRE as a test for the justifiedness of beliefs, not a method that citizens must actually apply in their belief dynamics. Thus, since the public political culture plays its role in the generation of citizens’ beliefs, but I am concerned with using MRE as a test for the justifiedness of the beliefs, I will not model a public political culture when simulating equilibration processes.

Nevertheless, one might say that sharing a political culture is relevant not only for the genesis but also for the justification of the citizens’ beliefs. For example, sharing a political culture might lead to the citizens’ considered judgments being similar in a certain way. This would be relevant for the justification of belief systems, since considered judgments are an important reference point for equilibration processes, both actual and hypothetical. This can be easily modelled, though the study presented below does not have this feature. In Sect. 11, I present a suggestion for capturing this aspect in future studies.

I have explained why the Rawlsian concepts of public reason and public political culture will not be in focus in this paper. Now let’s turn to what *will* be in focus, namely his claim that comprehensive doctrines need to support the political conception. Structurally speaking, there seem to be three possible connection types between a comprehensive doctrine and a political conception: support, incompatibility and neutrality. (Later I will introduce more, but these are much less relevant if at all.) It is clear that according to Rawls a comprehensive doctrine that is *incompatible* with a political conception cannot be part of an overlapping consensus on that conception. The whole point of reformulating JF is to make it compatible with various comprehensive doctrines.

In most relevant passages on the structure of overlapping consensuses, Rawls seems to think of comprehensive doctrines as *supporting* the political conception (e.g. PL xviii). Of course, in the above quote about the genesis of an overlapping consensus he writes that many citizens may come to affirm the political conception without seeing a connection to their comprehensive doctrine. But, again, there he is concerned with genesis and not justification. Only a few pages later in the same lecture (Lecture IV, §6 “Conception and Doctrines: How related?”), Rawls gives a model case with three comprehensive doctrines, each of them supporting the same liberal political conception of justice in their own way. Also, when explaining why an overlapping consensus is not a mere *modus vivendi*, Rawls stresses that in an overlapping consensus citizens are *morally* justified in endorsing the political conception, in contrast to the *pragmatic* justification of a *modus vivendi*. And he seems to think that the only way of doing so is that citizens “start from within their own comprehensive view and draw on the religious, philosophical, and moral grounds it provides” (PL 147). Thus, it is clear that Rawls thinks that in a well-ordered society, where citizens in an overlapping consensus are (morally) justified in endorsing the political conception, comprehensive doctrines must support the political conception.

Rawls seems to take this for granted, but I think it more appropriate to view this as a hypothesis. In terms of MRE, Rawls’s claim amounts to saying that in the endpoint of any equilibration process the following holds: If the citizen endorses both a comprehensive doctrine and a political conception, then the doctrine supports the conception. It is not obvious that this hypothesis is true. For example, even if a citizen’s doctrine is only partially comprehensive and silent about constitutional essentials (i.e., neutral about the conception), then it is offhand possible that they still endorse the political conception for its own merits, so to speak. In fact, it might even be possible for a citizen to tolerate a ‘mild’ or ‘local’ incompatibility between their doctrine and the political conception and still be in reflective equilibrium. These are relevant scenarios: The requirement that all comprehensive doctrines in a society need to support the political conception seems to be quite demanding. Rawls’s hypothesis poses a high standard that is not easily met. This is the problem in Rawls’s Political Liberalism I wish to contribute to: Which inferential connections between comprehensive doctrines and political conception make an overlapping consensus (in which citizens are fully justified in the above sense) possible or probable? In Sects. 8 and 9, I generalise these considerations and present a simulation study that addresses this question.

Let’s recap this section. First, the research question stated above (“Under which conditions are citizens in a society likely to be in full RE?”), is to be understood as a question about what Rawls calls *full justification*, i.e. the justification of the political conception as a part of the citizen’s belief system as a whole. In particular, it is not about the two kinds of justifications of the public sphere, *pro tanto* and public justification, which seem to presuppose society-wide full justification. Thus, public reason will not itself be represented, but only as a part of the political conception. Second, the research question is not at all concerned with the genesis of the citizens’ beliefs, but only with their justification. I interpret MRE as a test for the justifiedness of beliefs, not as the (only) method for generating justified beliefs. As a consequence, the important role of the public political culture in the genesis of an overlapping consensus is not modelled. Third, this research aims to contribute to the question of which (combinations of) inferential connections between the comprehensive doctrines and political conception in a society are compatible with there being a fully justified overlapping consensus. Rawls’s hypothesis that all comprehensive doctrines need to support the conception poses a high standard. The present simulation study is designed to address this hypothesis (see Sects. 8 and 9).

## The theory of dialectical structures

Beisbart et al. (2021) present a formal model of MRE. The central goal of this paper is to present a way of applying this model to answer the open question in PL. The next two sections give a short introduction to the model. It is based on the theory of dialectical structures by Betz (2010). In this section, I explain the fundamentals of this theory.

A dialectical structure is supposed to represent the state of a debate concerning a certain subject matter. Formally, each such structure is an ordered pair of two sets:

- A *sentence pool* *S*, representing the subject matter. This set of sentences is closed under negation (with $\lnot \lnot s := s$).
- A set of *arguments* *A* *on* *S*, representing the deductive relations between the sentences. Each argument is an ordered pair of a set of premises from *S* and a conclusion from *S*.

Any subset of *S* is a *position*. This subset represents the sentences that the agent accepts. To reject a sentence means to accept its negation. Here is an example for such a structure and some positions on it:

$$\begin{aligned} \begin{array}{ll} \begin{array}{l} S = \{\ s_1,\ s_2,\ s_3,\ \lnot s_1,\ \lnot s_2,\ \lnot s_3\ \}\\ A = \{\ \langle \{\ s_1\ \},\ \lnot s_3\rangle ,\ \langle \{\ s_2\ \},\ s_3\rangle \ \}\\ \end{array} &{}\quad \begin{array}{l} P_1 = \{\ \lnot s_1\ \}\\ P_2 = \{\ s_2,\ s_3,\ \lnot s_3\ \}\\ P_3 = \{\ s_1,\ s_2,\ s_3\ \}\\ P_4 = \{\ \lnot s_1,\ s_2,\ s_3\ \} \end{array} \end{array} \end{aligned}$$

We say that a position is *complete* iff it contains each sentence or its negation (or both), otherwise it is *partial*. $P_1$ and $P_2$ are partial, $P_3$ and $P_4$ are complete. We say that a position is *minimally consistent* iff it does not contain both a sentence and its negation. All above positions except $P_2$ are minimally consistent. A more robust notion is called *dialectical consistency* or simply *consistency*. This notion is defined separately for complete and partial positions. A complete position is *(dialectically) consistent* iff the position is minimally consistent and, for every argument, if the position contains all premises, then it contains the conclusion. The consistency of partial positions is defined with reference to the consistency of complete positions: A partial position is (dialectically) consistent iff the position is extended by some complete consistent position, i.e. is a subset of it. Positions $P_1$ and $P_4$ are consistent, $P_2$ and $P_3$ are inconsistent. (A position is *inconsistent* iff it is not consistent.) Lastly, the *content* of a consistent position *P* is represented by the intersection of all consistent complete positions that extend *P*. This intersection is again a position (denoted ${\overline{P}}$) and contains all sentences that the original position *implies*. An example: The content of $P_5 := \{\ \lnot s_3\ \}$ is $\overline{P_5}=\{\ \lnot s_2,\ \lnot s_3\ \}$.

This concludes my introduction to the theory of dialectical structures. Before I go on, however, let me stress how much hinges on *adequately* representing a dialectical situation. This includes the logical relationships between the sentences in *S*, because they need not be atomic. For example, $s_2$ could represent “The sun is bright” while $s_3$ represents “The sun is bright and it is warm”. In this case, the above set of arguments *A* is an *inadequate* representation of the deductive relations between the sentences, because it says that $s_3$ follows from $s_2$ even though it is the other way around. This potential mismatch is the price for the liberty one has when modelling a dialectical situation. The advantage is that the theory of dialectical structures is compatible with many different systems of logic. In what follows, I always assume that the dialectical situation is adequately represented.

## A model of reflective equilibrium

Based on these notions from the theory of dialectical structures, Beisbart et al. (2021) present a formal model of the method of reflective equilibrium. The dialectical structure (*S*, *A*) is assumed to be given and fixed. It represents the subject matter (including deductive relations) about which the agent is reflecting. At any time, the agent’s epistemic state can be represented by a pair of positions (*C*, *T*) where *C* is called the *commitments* of the agent (has to be minimally consistent) and *T* is called the *theory* of the agent (has to be consistent). The sentences in *T* are called the theory’s *principles*. This pair of positions mirrors the two components in Rawls’s presentation of MRE: the commitments correspond to the considered judgments, the theory corresponds to, well, the theory, and the theory is supposed to somehow match and account for the commitments. Concerning the equilibration process, the idea is again similar to Rawls’s: starting from some initial commitments $C_0$, we go back and forth between theory and commitments and make adjustments that improve the epistemic state until no further improvement is possible.

To make sense of this notion of improvement, the model defines an *achievement function*:

$$\begin{aligned} Z(C,T|C_0)\ =\ \alpha _A\cdot A(C,T)\ +\ \alpha _S\cdot S(T)\ +\ \alpha _F\cdot F(C|C_0) \end{aligned}$$

This real-valued function is the sum of three inner functions with weights $\alpha _A+\alpha _S+\alpha _F=1$. The inner functions represent desiderata for epistemic states:

- *Account*, *A*(*C*, *T*): measures how close the current commitments *C* are to the content ${\overline{T}}$ of the current theory *T*. This is supposed to reflect how well the theory matches the commitments and accounts for them.
- *Systematicity*, *S*(*T*): measures how systematic the current theory *T* is. Less principles and more content improve this function. This desideratum captures the idea that we want a theory to systematise our commitments. Without this desideratum, we could just set $T:=C$, as long as *C* is consistent.
- *Faithfulness*, $F(C|C_0)$: measures how close the current commitments *C* are to the initial ones $C_0$. The idea here is to have some tie to the starting point such that an agent cannot without good reasons discard the commitments they started with.

For the mathematical definitions, see the Appendix. The weights $\alpha _A,\alpha _S,\alpha _F$ make the trade-off between desiderata explicit. As a standard configuration, $\alpha _A=0.35$, $\alpha _S=0.55$, $\alpha _F=0.1$ has proven to yield plausible results. For a more detailed exposition and motivation of the achievement function, see Beisbart et al.’s (2021) paper.

It should be noted that the achievement function does not aim for ‘mere consistency’ or a ‘mere match’ between theory and commitments. Instead, it aims for coherence. The desideratum of systematicity urges the agent to choose a theory that *systematises* the commitments and in this sense explains them, makes sense of them, or helps us understand them. Importantly, the relation of explaining or making sense does not appear in the structure itself which only contains inferential relations. Instead, this relation is more of a macro feature that obtains between two positions, namely a highly systematic position (the theory) the content of which matches another position (the commitments).

Given the above definition of achievement, the *algorithm* of MRE is defined as follows. Let dialectical structure and initial commitments $C_0$ be given. Now, out of all consistent positions on the structure, a theory $T_1$ is chosen that maximises the achievement function for the initial commitments, i.e. maximises $Z(C_0,T|C_0)$. If two or more score best, we make a random choice between those. Then, we adjust the commitments: Out of all minimally consistent positions, a new set of commitments $C_1$ is chosen that maximises the achievement function for the current theory, i.e. maximises $Z(C,T_1|C_0)$ (again, we make a random choice in case of a draw). We then go back and forth, holding the commitments (or theory) fixed while maximising achievement by chosing a new theory (or new commitments). This goes on until no adjustment of either theory or commitments improves achievement anymore: we have reached an *equilibration fixpoint*.

The definitions of achievement function and algorithm together yield a precise specification of the method of reflective equilibrium. Given a subject matter (i.e. dialectical structure) and initial commitments (i.e. a position), it says how exactly adjustments are made and when an end state (i.e. fixpoint) is reached. In fact, the model can be programmed such that computers can run equilibration processes. Of course, this model is not the only plausible specification of MRE. For example, it is possible to conceive of more or different desiderata. Also, the mathematical definitions of these functions, particularly the specific values of the various weights in them, are to some extent arbitrary (as is to be expected). It is possible that both the achievement function and algorithm will be superseded by more plausible or more elaborate versions.

However, as a deliberately simple starting point, the model seems plausible enough, especially since it has undergone quite thorough testing. In their 2021 paper, Beisbart et al. discuss some equilibration processes on a specific example structure. They argue that the results are plausible and match our pre-theoretic expectations of MRE (2021, Sect. 3). Also, they prove some basic analytic results that lend further plausibility to the model (2021, Sect. 2.4). In addition, the research group around Beisbart et al. (of which I myself am a member) has assessed the model by running simulations on large sets of randomly generated structures and analysing the results. The findings are publicly available in a recent technical report by Freivogel and Cacean (2021). It seems to me that this analysis corroborates the model, at least for the large part. This is not to say that it’s all done and dusted. But it warrants treating the model as a starting point for further research: applying it to interesting scenarios, examining variations and extensions, etc. In fact, one such variation is better suited for the present study than the original model, as I argue next.

## Changing the model: bounded rationality

In this section, I motivate changing the algorithm to a locally optimising one. First, I distinguish two views one can have on the nature of MRE, namely proceduralism and consequentialism, and opt for the latter. Then, I define a local variant of the algorithm and explain why it is more suitable for addressing my research question given a consequentialist view.

It is possible to specify method and state of reflective equilibrium independently from each other. In that case, the result of the method (i.e. the fixpoint) might not be a state of reflective equilibrium. For example, we might require for a state of reflective equilibrium that the state is *globally optimal* according to the achievement function, i.e. there is no epistemic state with higher achievement. (Of course, any fixpoint is *semi-globally optimal*, because any *separate* adjustment of either theory or commitments will decrease achievement. However, it is still possible that some *concurrent* adjustment of both theory and commitments improves achievement.) Moreover, we might also require that theory and commitments *match perfectly*, i.e. that $C = {\overline{T}}$. (If an epistemic state fulfills both requirements, Beisbart et al. call it a “full RE state” (2021, p. 449). Here the term is reserved for what Rawls meant by it.) Given the above definitions of algorithm and achievement function, not all fixpoints are RE states in this demanding sense and *vice versa*. This raises interesting questions about the relation between algorithm and achievement function. Both seem to give independent verdicts on what epistemic state should be adopted. Which has more authority?

Proceduralism will say that the algorithm has ultimate authority. The achievement function is merely a tool helping in the process. It doesn’t matter what it says about states that are not considered during the process. Consequentialism, on the other hand, will say that the achievement function has ultimate authority by giving an axiology for epistemic states. For example, one could say that it represents the degree to which a state is in equilibrium, a feature that is deemed exclusively epistemically valuable. The algorithm is then simply a means to an end, increasing epistemic value. I am leaning towards consequentialism, though I will not argue for it here and simply presuppose it for the purpose of the paper.

Given that the algorithm is just a means to an end, we are not strictly bound to a particular version of it. For example, instead of using the semi-global algorithm from the last section, we could opt for a global one: Calculate achievement for *all* combinations of commitments and theory and choose (one of) the best one(s). Or, quite the contrary, we could stick to the step-wise adjustment of the semi-global algorithm, but optimise *locally* by looking for the best commitments in the close neighborhood of the previous commitments, likewise when adjusting the theory. This kind of piece-meal change is most likely what Goodman, one of the earliest proponents of MRE, had in mind (1955, p. 67). Since I am going to opt for this kind of local algorithm, let me give you a detailed definition.

The basic idea is to change only single sentences. That is, the set of candidate commitments in any adjustment step contains all minimally consistent positions that *either*

- extend the current commitments by any one sentence (negations included), *or*
- are extended by the current commitments by any one sentence (negations included), *or*
- result from removing any one sentence (negations included) from the current commitments and adding that sentence’s negation (with $\lnot \lnot s := s$).

Now, we calculate the achievement of these candidate commitments and choose the best one (selecting at random in case of a draw). For adjusting the theory, we proceed exactly the same way except requiring *(dialectical) consistency* instead of only minimal consistency. Note that we start out with a set of independently given initial commitments, so we can construct the first set of candidate commitments from these. We do not have such an independently given first theory. Thus, we must define some such theory and do so by setting $T_0 := \emptyset$. There are other ways of defining $T_0$, but in the interest of keeping it simple (and feasible), the empty set seems a plausible enough starting point.

For my purposes, this local algorithm is more suitable than the semi-global or global one. There are several reasons for this. First, note that the local algorithm is good at reaching fixpoints with high achievement. Flick (2022) has tested the local algorithm and compared it to the semi-global one. His general upshot is that the local algorithm is as good as the semi-global one. However, this result only holds in structures with one-premise-arguments. This is one of the reasons why the study design (Sect. 9) features only one-premise-arguments. (Presumably, the local alogrithm would have to consider changes to more than one sentence per step in order to work for arguments with more than one premise.)

Second, the local algorithm is much more feasible than the more global versions. To see this, consider an unrealistically small sentence pool of size 40 (including negations). This already yields $3^{20} \approx 3.5$ billion minimally consistent positions (i.e. commitments candidates). The semi-global algorithm requires going through all of them and calculating which most improves achievement. This requires crazy computational resources. Even the most advanced computers fail at this, let alone human brains. This makes it unsuitable for the present research. Not only, because the simulations could not be run. More importantly, as I already pointed out in Sect. 4, the Rawlsian solution to the problem of reasonable pluralism fails if it turns out that a full RE is unrealistic or even impossible. The goal is to find full RE conditions for real (or at least realistic) societies with epistemically non-ideal citizens. Not much is gained if we find conditions that only hold for currently unavailable supercomputers with extreme computational power. In short, I embrace a *bounded rationality* perspective. Instead of making the usual strongly idealising assumptions, I try to stay closer to the cognitive capacities of actual citizens. The local algorithm is suitable for this aim. In the above example, we now only have 40 instead of 3.5 billion commitments candidates in each step. To be sure, it would still be a lot of work for a human brain and it is an open question how feasible it really is. Nonetheless, it is a simple and initially plausible idea for an algorithm that has at least a chance of being feasible for us. For these reasons, I think the local algorithm is the right choice for researching the open question about full RE in liberal democracies.

Before connecting the formal apparatus to my research question in a more detailed manner, let me sum up this section. Beisbart et al. have put forward a formal model of reflective equilibrium. It consists of two parts, the achievement function and an algorithm. The model has been tested, with good results. However, the semi-globally optimising algorithm is computationally demanding. In a non-ideal setting, which I am embracing, a locally optimising algorithm is the better choice. The local algorithm has also been tested with promising results. I think that the current status of the model warrants application to interesting cases like Rawlsian full RE. This application will be a two-way learning process. By applying a plausible specification of MRE to the scenario, we learn something about the scenario. But we also learn something about the model: If it yields utterly implausible results, then this might incentivise us to change it. If it doesn’t, however, then the fruitful application corroborates the model further. The present paper aims to start this two-way learning process.

## The question recast

In Sect. 3, I pointed out that the following is an open question in Rawls’s PL:

*Under which conditions are citizens in a society likely to be in full RE?*

In principle, it would be interesting to study the influence of all kinds of conditions: socio-economic, political, technological, etc. My focus will be on the epistemic kind, more precisely, on epistemic conditions that can be encoded using the model described in Sect. 6. The goal of this section is to recast the open question from PL in terms of the model.

Let’s start with the notion of a wide and general, or full, RE (PL 384n).

- *Wide RE*: For a citizen to be in wide RE, they must have carefully considered alternative conceptions and their arguments and not just ‘smoothed out’ some irregularities in their own judgments. Obviously, it is too much to ask that they consider all logically possible statements on the subject matter, but we might expect them to consider at least the most important proposals that are discussed in their society (cf. TJ 43). My suggestion for representing this in the model: We require that all citizens in a society run their RE processes on a shared dialectical structure. That way when making adjustments all will consider the same set of sentences and the same set of arguments.
- *General RE*: For the citizens in a society to be in general RE, they must affirm the same political conception of justice (e.g. JF) in their considered judgments after reaching RE. My suggestion for representing this in the model: A particular sentence is marked as a political conception of justice (PC). An RE is general about PC if PC is an element of all (or most) citizens’ fixed point commitments. Alternatively, we can mark a subset of the sentence pool *S* as alternative political conceptions of justice. RE is general if there is a particular one of them that is an element of all (or most) citizens’ fixed point commitments.

To sum up, citizens in a society have reached full RE iff, after running their RE processes on a shared dialectical structure, all (or most) accept the same political conception of justice in their fixed point commitments. Note that the requirement that citizens share the dialectical structure is a very basic publicness condition. Of course, it falls short of fully capturing the influence of a public political culture (see Sect. 4), but it goes a step in that direction by excluding the possibility that citizens do not even consider the same set of sentences and arguments in MRE.

Which aspects bear on the likelihood of a full RE? Given the model, there are two, maybe three:

- the dialectical structure of a given society: which comprehensive doctrines and commitments are up to debate in a society.
- the citizens: especially initial commitments, but also random choices that are made during equilibration.
- (the achievement function and algorithm: e.g. how the three parts of the achievement function are weighted, or what to do in case of a draw when adjusting commitments or theory. I assume this to be fixed, but in principle, this may vary too, between societies as well as between citizens.)

In my study I wish to focus on the first of these. That is, I wish to study which kinds of dialectical structures make a full RE more likely than others. More precisely, as I already explained in Sect. 4, I am interested in the influence of the inferential relationships between the political conception(s) and the comprehensive moral doctrines that are up to debate in a society. Rawls supposes that all comprehensive doctrines must support a political conception. Otherwise, the conception cannot be the focus of an overlapping consensus for the right reasons, or so he assumes. This is a hypothesis worth testing, as I argued earlier. Thus, my guiding question for the study is:

*Which kinds of inferential relationships between the political conception(s) of justice and the comprehensive moral doctrines make a full RE more likely than others?*

Before I go on to present the design of a first study, it is worth noting that this research question is general in an important sense. It does not ask whether a consensus on Rawls’s own proposed political conception (JF) in particular is likely or not. Rather, it asks what structural conditions *any* political conception of justice needs to satisfy in order to more likely be agreed upon. Whether JF (or any other political conception) *in particular* satisfies these conditions in some existing (or ‘realistic’) societies is an additional question that needs further, perhaps empirical, argument. For now, let’s start investigating on the very general level.

## Design of the study

**Fig. 1:**
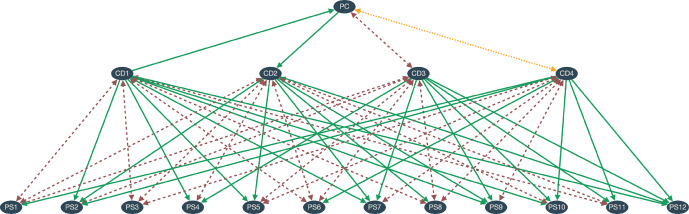
Solid green arrow = implication, dashed red arrow = incompatibility, dotted yellow arrow = joint exhaustiveness. This scheme shows the 17 sentences (without negations) and examples for arguments between them satisfying conditions 1–9. Note that the arrows can represent connections to negations of sentences even though the negations themselves are not shown in the scheme. Also note that incompatibility and joint exhaustiveness are symmetrical by contraposition. Due to conditions 5, 7 and 9, the set of arguments in each dialectical structure of this class can be divided into two parts: a head (visually above the CDs) and a body (visually below the CDs)

We consider a class of dialectical structures and sets of initial commitments satisfying the following conditions (also see Fig. 1):

1. The sentence pool consists of 17 sentences plus their negations, i.e. $|S| = 34$.
2. The set of arguments contains only arguments with one premise.
3. The set of arguments is such that there is at least one complete consistent position.
4. We separate the sentences (without negations) into three classes of statements:
  - there are 4 comprehensive doctrines (CD1–4), each a general statement or conjunction of general statements,
  - there are 12 particular statements (PS1–12), and
  - there is 1 political conception of justice (PC).
5. Any PS can only follow from or be incompatible with each CD. There are no other arguments connecting both classes. This follows from the idea that comprehensive doctrines are general moral theories.
6. The set of initial commitments is always a subset of the particular statements (and their negations).
7. The CDs are somewhat independent from each other, there are no direct inferential relationships between them. Note, however, that they will usually be pairwise incompatible due to one implying and the other denying some particular statement.
8. PC can have any kind of inferential relationship with each CD.
9. There are no direct inferential relationships between PC and the PSs. The PSs are supposed to be everyday life moral judgments and as such somewhat independent from the political sphere. In other words, the particular statements of the politicial sphere are not represented.

Obviously, these are quite restrictive conditions. In particular, in future studies it might be interesting to soften up conditions 1, 2, 4, 6 and 9 to get more general classes of structures. Also, note that there are no structural alternatives to PC: In societies with this kind of structures, there is only one available political conception. Also, note that the initial situation or particular descriptions of it, like the original position, are not modelled. This is because the initial situation does little justificatory work. It is unclear whether it should be modelled in later versions.

The main advantage of the present design is its simplicity. As Hegselmann and Krause have repeatedly shown in their work on opinion dynamics (e.g. 2002), modelling with the KISS-principle (“Keep it simple, stupid!”) is a valuable research approach (ibid, p. 9). Even unrealistically simple models can uncover how interesting (and real) mechanisms work. The results can serve as a benchmark for more complex models. As we will see in Sect. 10, the simplicity of the model will enable us to clearly understand the results. This step (finding simple, well understood and plausible examples) is a crucial step whenever employing a new strategy. If it is successful, as I argue later it is, then the strategy is validated for the time being and we can introduce more complexities.

The kind of structures resulting from the above conditions is visualised in Fig. 1 with possible inferential connections. Given this visualisation, let’s call the set of arguments connecting PC and the CDs the structure’s *head*. The set of arguments connecting the CDs and the PSs is called the structure’s *body*. We can now rephrase the guiding question for this study more precisely:

*Which kinds of heads make a full RE more likely than others?*

Before having a closer look at the heads, let’s talk about the bodies and the initial commitments. Since we are here only interested in the influence of the heads, we will randomise both. For each pair of CD and PS, there are three possibilities: no connection, the CD implies the PS, or they are incompatible. By going through all these pairs and realising any of the three possibilities with an equal probability of 1/3, we can generate bodies at random. The initial commitments are also generated at random with a homogeneous probability distribution.

Let’s have a closer look at the heads. For each of the four CDs, there are five connection types that the CD can have to the PC:

1. No: There is no inferential connection to the PC.
2. Follows: CD follows from PC / PC implies CD.
3. Implies: CD implies PC.
4. Denies: CD denies PC / they are incompatible / CD implies the negation of PC (and *vice versa*).
5. Or: CD and PC are jointly exhaustive / the negation of CD implies PC (and *vice versa*).

Given the interpretation of the CDs as comprehensive moral doctrines and PC as a political conception of justice, not all of these connection types make sense. In particular, it is hard to think of a pair of comprehensive doctrine and political conception exhibiting the Follows connection or the Or connection. I have not managed to come up with respective examples. Perhaps these connection types are impossible on conceptual grounds. However, to keep things general for now, I include them.

How many heads are there? With four CDs, each having one of five possible connections to PC (repetitions allowed), there are $5^{4}=625$ possible 4-tuples of the five connection types. However, it’s not necessary to run simulations on the full spectrum of these. Consider, for example, the tuples (Implies, No, No, No) and (No, Implies, No, No). For my purposes, there is no relevant difference between these. It doesn’t matter *which* comprehensive doctrine has a certain connection type, but only *how many*. In mathematical terms, I am interested in all 4-multisets of the five connections types. There is a total of 70 such multisets. That is, there are 70 heads.

Now, for each of these 70 heads, 100 random bodies are generated. For each of the 7.000 resulting structures, 10 random initial commitments are generated. Thus, I will run a total of 70.000 equilibration processes.

We can then for each head calculate the percentage of processes that lead to acceptance of PC in the fixed point commitments. Let’s call this the *acceptance rate* of PC. If this percentage is higher for a certain head than for others, then this head makes acceptance of PC more likely than others. Also, we can partition the set of heads into interesting classes. For example, we can count how often heads contain the inferential relationship Implies. This gives a partition of 5 cells: the cell containing all heads with no/one/two/three/four occurences of Implies. (The last cell containing only one head, of course.) We can then for each cell calculate the average acceptance rate of PC. If the average rate gets higher with more occurences of Implies, then this inferential relationship seems to promote acceptance of PC. If it gets lower with more occurences, then Implies seems to counteract acceptance of PC.

In the next section, I will present some of these results. But first, two important disclaimers about acceptance rates: First, they only tell us something about the probability that a randomly drawn RE process will lead to acceptance of PC. But it does not give us directly the probability that all or most citizens in some society will *agree* on PC after equilibration (which is the condition for full RE). However, if a certain kind of head makes acceptance of PC more likely for a particular RE process, then consensus on PC in a society with that kind of head is more likely as well. Thus, studying acceptance rates also contributes to answering the guiding question: *Which kinds of heads make a full RE more likely than others?*.

The second disclaimer is that even if we find interesting structural features that boost acceptance rates, this does not yet fully answer the challenge of reasonable pluralism, for two reasons. First, as already noted at the end of Sect. 8, further argument is needed to show that it is realistic that JF or some other viable political conception of justice can satisfy these conditions in existing societies. Second, even if acceptance of PC is high, it might be that this comes at the expense of pluralism. That is, there might be structures that foster consensus *by* diminishing pluralism. But we are interested in consensus that comes about *despite* pluralism. Further studies will have to focus on this point. Here I only consider the influence on acceptance rates.

With these qualifications in mind, let’s have a look at some results.

## Results

We start with some interesting mean values (always averaging over all heads, standard deviation in parentheses). The average percentage of processes that lead to acceptance of PC in the fixed point commitments, i.e. the average acceptance rate, is 34.7% (37). PC almost never occurs in the fixed point theory, only in an average of 0.3% (0.6) of the processes. What about the four CDs? Given their relation to the particular statements (only implying or denying them, thus being ‘general’ in this sense), we expect them to often occur in the fixed point theory even though they never occur in the initial commitments. And, indeed, in 57.8% (26.4) of processes, there are only CDs in the fixed point theory. In 37% (28.7) of the processes, there are only particular statements in the fixed point theory, and in the remaining 5.2% (2.7) of cases, the fixed point theory contains both. All these numbers have significant standard deviations (averaged over the heads). This suggests that the heads have a strong influence on these percentages. Let’s have a look at the influence on the acceptance rate of PC.

I wish to focus on how often the different kinds of connections between the CDs and PC occur, just like I sketched at the end of the last section. That is, for each of the five connection types, we partition the set of heads into five cells containing all heads with no/one/two/three/four occurences of that connection type. For each cell, we can average the acceptance rate over all heads in the cell.

**Table 1:** This table summarises some information about the 2-cell of the Implies-partition

Head	# No	# Follows	# Implies	# Denies	# Or	Acceptance rate
h_10	2	0	2	0	0	32.7
h_20	1	1	2	0	0	2.4
h_27	1	0	2	1	0	33.8
h_28	1	0	2	0	1	47.3
h_40	0	2	2	0	0	1.7
h_47	0	1	2	1	0	4.3
h_48	0	1	2	0	1	40.5
h_59	0	0	2	2	0	37.6
h_60	0	0	2	1	1	45.3
h_61	0	0	2	0	2	97.9
					Average	34.4

The rows reference the ten heads in this cell. The columns reference the number of occurrences of the five connection types. The last column displays the acceptance rate of each head

Let me give you an example to make this idea a little more tangible. Consider the 2-cell in the Implies-partition, i.e. the cell containing all heads in which exactly two CDs imply PC. Table 1 lists these heads together with their acceptance rates. The cell contains all of the ten possible combinations of the other four connection types for the remaining two CDs. Thus, if we calculate the average acceptance rate of PC in this cell, we average over all differences that other connection types make. Note that each head in this cell occurs in other cells as well. For example, head h_27 occurs in the 1-cell of the No-partition, the 0-cell of the Follows-partition, the 1-cell of the Denies-partition and the 0-cell of the Or-partition.

**Fig. 2:**
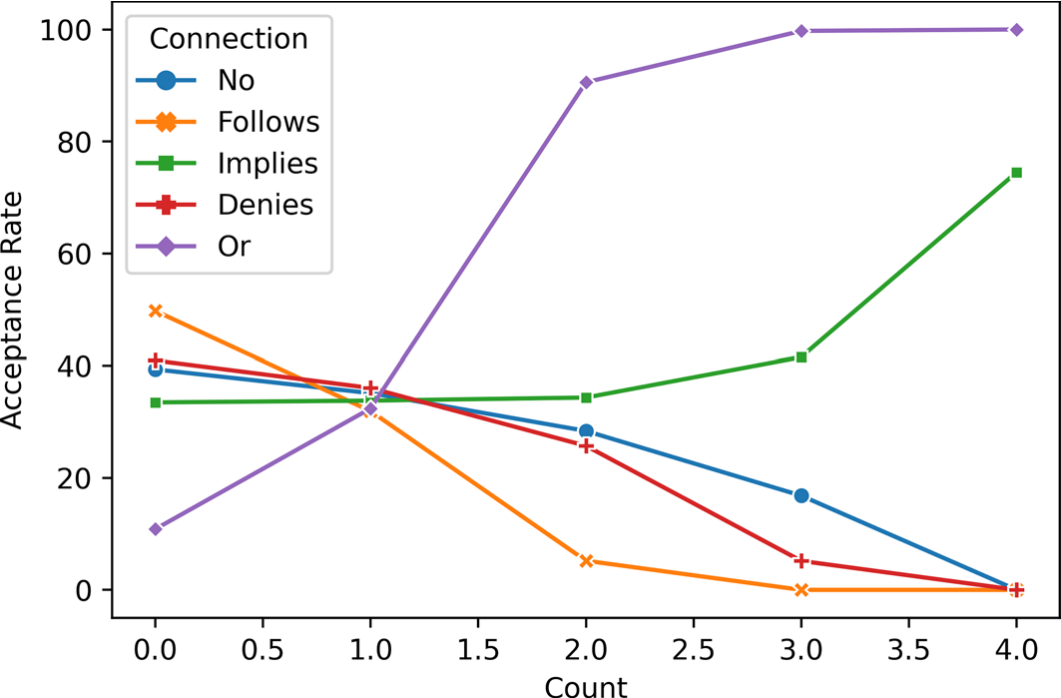
The table above shows the average acceptance rates of PC in all cells of all partitions. The rows reference the partitions, the columns reference the cells in the partitions. The lineplot below the table visualises that data

The results for all partitions are shown in Fig. 2, consisting of a table and a lineplot visualising the data in the table. First, note that for each of the five connection types, the average acceptance rates are an either monotonically increasing or monotonically decreasing function of the number of occurences of the type. This suggests that every connection type either *generally* promotes or *generally* counteracts acceptance of PC. Note also that all connection types have a noticeable impact. The No-type has the smallest range of average acceptance rates: It ranges from 39.4% if it does not occur (meaning all principles have some inferential connection with PC) to 0.0% if it applies to all principles (meaning that no principle has an inferential relationship with PC). All other types have an even greater range. The Or-type takes the win in this regard ranging from 10.8% (no occurences) to 100.0% (four occurences).

We can classify the connection types into two groups. No, Follows and Denies seem to counteract acceptance of PC: average acceptance rates drop with more occurences of these three types. Implies and Or, on the other hand, seem to promote it: average acceptance rates rise with more occurences of these two types. Can we rank the types within both groups by how strongly they counteract or promote PC? It seems we can, at least for the large part. Follows counteracts PC the most: with 49.8% it has the highest average acceptance rate for no occurences. From there it drops quickly to 5.2% for two occurrences. If it occurs three or four times, the acceptance rate is Zero (meaning that not a single RE process leads to acceptance of PC anymore). No and Denies are pretty comparable. They drop from about 40% for no occurrences down to Zero for four occurrences. Perhaps No fares a little better, since it has noticeably higher average acceptance rates for two and three occurences. Turning to the connection types that promote acceptance of PC: Or clearly takes the win with the lowest acceptance rate of 10.8% for no occurences going up to around 100.0% for three and four occurrences. Implies promotes PC less strongly starting from 33.5% for no occurrences going to a maximum of 74.5% for four occurences. Table 2 gives an overview of these considerations.

**Table 2:** This table summarises a first interpretation of the results by roughly categorising the influence that the five connections types (referenced by the rows) have on acceptance of PC

	Form	Influence	
No	x	−	Moderately negative
Follows	$\lnot$CD$\rightarrow \lnot$PC	$- -$	Strongly negative
Implies	CD$\rightarrow$ PC	+	Moderately positive
Denies	CD$\rightarrow \lnot$PC	−	Moderately negative
Or	$\lnot$CD$\rightarrow$ PC	++	Strongly positive

The column labelled ‘Form’ displays a certain kind of argument form that each connection type (save the No-type) can be displayed as. It might help explain the results, see the discussion of this table

Let’s set aside the connection type No and focus on the remaining four types. In table 2, I have indicated a particular argument form that each connection type can take. The form is always that of an implication with a CD (or its negation) as the antecedent and PC (or its negation) as the consequent. This gives us two distinctions for the four connection types. First, we can distinguish according to whether the antecedent is CD or its negation. Second, we can distinguish according to whether the consequent is PC or its negation. You may have noticed that we can connect these distinctions to the influence that the connection type has: If the antecedent is CD itself, the influence is moderate. If it is CD’s negation, the influence is strong. If the consequent is PC itself, the influence is positive. If it is PC’s negation, the influence is negative.

The latter is to be expected, since we are studying the influence of the connections between CDs and PC, and RE processes are likely to lead to acceptance and/or rejection of at least a part of the CDs. Connection types that imply PC upon acceptance (or rejection) of a CD promote acceptance of PC, because there is a chance that during equilibration the agent accepts (or rejects) the CD, in which case the RE process will incentivise the agent to also accept PC. (This is because PC is in the content of the CD (or its negation) and the agent may shorten the distance between her commitments and the content of her theory by accepting PC, thereby promoting account, thereby promoting achievement, see Sect. 6.) Likewise, if the negation of PC is implied by a CD (or its negation), it will be more likely that the agent will reject PC and, because her commitments ought to be minimally consistent, not accept PC.

This explains why the four connection types either promote or counteract acceptance of PC. What about the strength of this influence? It seems that implications of the negation of a CD have a stronger influence than implications of a CD itself. It is not obvious why this is the case, but a natural conjecture is that CDs are more likely to be rejected than accepted during RE processes. Then for any occurence of Follows and Or there is a higher chance that the antecedent will be accepted during equilibration compared to an occurrence of Implies or Denies. In turn, there will be a higher chance that the consequent will be accepted. In fact, the conjecture turns out to be true: On average, in 885 of the 1.000 processes per head, more CDs are rejected than accepted. (This is not surprising or implausible. Accepting some CD will likely lead to rejection of the others, because they are usually incompatible with each other due to giving conflicting verdicts on the PSs.) So this might explain the strength of the influence of the connection types.

This concludes my presentation of the results of the study. The main takeaway is summarised in Table 2. I have attempted a preliminary explanation of the results.

## Discussion and outlook

At last, I wish to briefly situate them in the bigger picture. What do the results tell us? I think they are relevant in two regards.

First, the results corroborate my research approach, because they are plausible. First, it was to be expected that most citizens after equilibration accept one of the comprehensive doctrines and reject the others. This is in fact what happened. Second, it was to be expected that the only reason for citizens to accept the political conception is its inferential connections to the comprehensive doctrines, because it is otherwise isolated and does not itself occur in the initial commitments. This expectation fits nicely with the fact that the No connection type negatively influences acceptance rates. Finally, the influence of the other four connection types also fits nicely with both expectations taken together, as I have argued at length above. Thus, the results are plausible. A substantially different picture might have raised the worry that something is fundamentally amiss either with how I represented the dialectical situation (conditions 1–9 in Sect. 9) or with the formal model of RE (including the local algorithm) or both. Thus, I take the results to show that the strongly simplified representation of this dialectical situation in a liberal democracy is a promising starting point for more elaborate study designs.

Second, even though the model is strongly simplified, the results are still informative beyond corroborating the general approach. If the simplified model shows a certain (plausible) behaviour, then this shows that more complex mechanisms are not strictly necessary for that behaviour to obtain. What is the relevant behavior here? If we ignore Follows and Or, because these relationships are unlikely, perhaps impossible, to occur between a CD and a PC, then we are left with No, Denies and Implies. (These correlate with neutrality, incompatibility and support, respectively, see Sect. 4.) Out of these three, only Implies promotes consensus. This is in line with Rawls’s hypothesis that comprehensive doctrines need to support the political conception. In particular, incompatibility makes a consensus impossible and neutrality is not enough either. It is interesting that this simple study design seems to verify Rawls’s hypothesis. And, since it sets a high standard, it is worrisome. In particular, I think it is worrisome that the No connection type fares so badly. Of course, it is well possible that these results are not robust when the design is de-idealised, see my suggestions below. Nonetheless, even if not robust, they are a good comparison point for future results. If future de-idealised designs significantly change these results, then the simple design is a useful benchmark to compare against. It can then be said precisely what the effects of certain changes are.

So let’s turn to possible de-idealisations and modifications of the study design. I think the most plausible next steps are the following three. First, the worrisome fact that the No connection type counteracts acceptance of PC is explained by the fact that the only incentive to accept PC is given by the inferential connection to a CD (and acceptance or rejection of that CD, of course). Thus, a natural starting point for de-idealisation is to add PSs to the ‘political’ part of the structure, i.e. add PSs that are implied or denied exclusively by the PC. The initial commitments, then, span not only the regular PSs, but also the PPSs (short for ‘political particular statements’). Since the initial commitments take a stand regarding the PPSs, there is an incentive to accept a PC that accounts well for these political initial commitments, because that increases account without decreasing faithfulness. However, it might decrease systematicity, if the resulting theory is a compound of CD and PC instead of a standalone CD. If equilibration processes end with such a compound theory of CD and PC, then I think it is a good representation of what I called ‘justifiedly accepting a PC for its own merits’ in Sect. 4. Importantly, this is possible even if one’s CD is neutral about the PC (*pace* Rawls). Of course, we do not know whether this will happen a significant number of times, but adding PPSs to the structures is, I think, the best candidate modification for challenging Rawls’s hypothesis.

Second, adding PPSs to the structures will open up the possibility of modelling the influence of a public political culture as I hinted at in Sect. 4. The fact that citizens live in a shared political culture might lead to them sharing the same or similar considered judgments regarding consitutional essentials. In terms of the model, the initial commitments of citizens in a society are the same regarding the PPSs and vary only regarding the PSs. Presumably, this will boost the acceptance rate for a PC to the extent that the shared initial commitments align with the content of that PC. Note that this should not be interpreted as citizens having the same considered judgments about all political matters, but only about constitutional essentials.

**Fig. 3:**
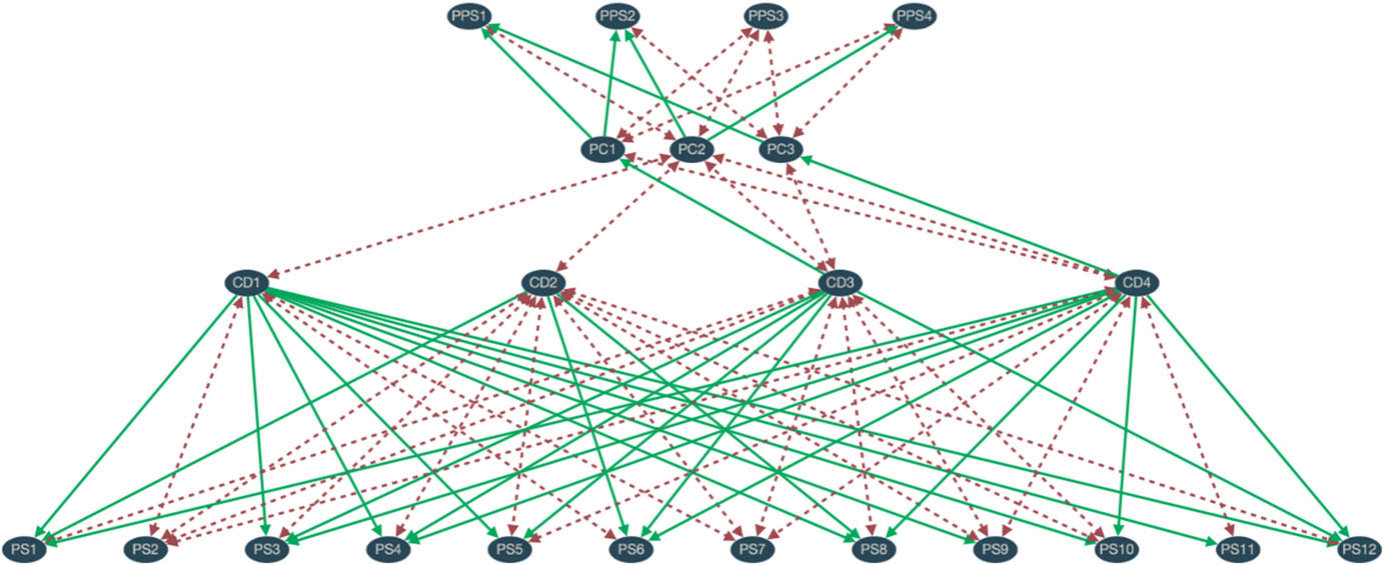
Example of a structure with three PCs and four PPSs, analogous to Fig. 1. Since the initial commitments take a stand regarding the PPSs, there is an incentive to accept a PC that accounts well for these political initial commitments. Here, the structure’s head is in the middle while the structure’s body is composed of the top and the bottom part together (the terminology is less intuitive here)

Third, a very important de-idealisation is to introduce structural alternatives to PC. That is, instead of only one political conception that is up for debate in each society, there are two or three rival ones. This is much more realistic and will presumably make convergence on any particular PC harder. It addresses the potential worry that the idealisation of the first study presented here leads to results that are too optimistic. If we take this and the preceding modifications together, then the structures will look like the one in Fig. 3.

In addition to these very next steps, there is a host of possible modifications, extensions and applications of this general modelling approach. Further modifications include adding more complex arguments, adding mid-level principles between CDs and PSs, allowing more or all sentences to be in the initial commitments, etc. A very natural and valuable extension of the model might be to add weights to the (initial) commitments. There are several possibilities for this, ranging from simple numerical representation to perhaps Muldoon et al.’s ideology attribute (2012).

Finally, the (modified and extended) model can be applied to empirical data. As of now, structures and initial commitments are randomly generated with homogeneous probability, thus, the findings hold for the possibility space as a whole. This by itself is, of course, a valuable insight. It helps us understand how MRE generally works given certain boundary conditions. If there is no defeating evidence, we can infer that MRE works similarly in real cases. This is not unlike the statistical inference from studies about drug efficacy to what can be expected in individual cases. Nonetheless, it is always possible that the more realistic subset of the possibility space as a whole shows a somewhat different behaviour, just like a certain class of individuals might react differently to a drug than the population as a whole. Thus, it will be worthwhile to conduct empirical studies about the following: First, what kind of structures underlie the dialectical situation in particular real societies? Second, what kind of considered judgments do real citizens have in these societies? We can then pair these empirical boundary conditions with simulations of MRE to see whether and how an overlapping consensus is possible. In principle, this can go both ways, we might find that a consensus is easier to achieve than in possibility space as a whole, or harder.

## Conclusion

In this paper I have outlined how a recently developed formal model of reflective equilibrium might be used to address an open question in Rawls’s *Political Liberalism*, namely “Under which conditions are citizens in a society likely to be in full RE?”. I have presented a first study that focuses on a certain kind of conditions concerning the inferential connections between the political conception of justice and the comprehensive doctrines in a society. The results suggest that acceptance of PC is promoted by the Implies-connection (CD implies PC) and the Or-connection (CD and PC are jointly exhaustive). The latter has a stronger influence than the former. On the other hand, the Denies-connection (CD and PC are incompatible) and the Follows-connection (CD follows from PC) counteract acceptance of PC, again the latter having a stronger influence than the former.

I have argued that these results validate the present strategy, because they are in line with expectations that are plausible given the modelling conditions. Additionally, they show that the assumptions of this study are sufficient for Rawls’s hypothesis that the comprehensive doctrines in a well-ordered society must support the political conception. This is a rather demanding standard and it would be worrisome if it turned out to be the only workable one. Of course, it is unclear whether this hypothesis also holds for more elaborate designs. Thus, the results should be treated as a benchmark for future studies. I highlighted three modifications as particularly important. First, political particular statements should be added to the structure so that a political conception can account for them. I have argued that this might challenge Rawls’s hypothesis. Second, the influence of a shared political culture can be represented by imposing a similarity condition for citizen’s political initial commitments. Third, adding structurally alternative political conceptions to the setup will make the model more realistic and addresses worries that the simplifications deliver results that are too optimistic.

As the designs get more complex, we better understand how the model works and how RE works in cases that are structurally similar to situations of reasonable pluralism. Piece by piece we might be able to uncover conditions that make an overlapping consensus for the right reasons more likely in real-world scenarios.

## Appendix: The achievement function

The achievement function is the weighted sum of faithfulness, account and systematicity. Let’s start with account: The basic idea is that a theory accounts for a sentence iff the sentence is in the theory’s content. Regarding sets of sentences, like an agent’s commitments, account is a matter of degree. Let $\langle S, a\rangle$ be a dialectical structure. A theory *T* accounts for some commitments *C* to the extent that its content ${\overline{T}}$ contains all of them and only them. Formally, we first measure the (weighted) Hamming distance between *C* and ${\overline{T}}$, and normalise it by the number of unnegated sentences $N:=|S|/2$. Then, we plug this distance into a monotonically decreasing function $G:\mathbb {R}\rightarrow \mathbb {R}$ to get the “closeness” of *C* to ${\overline{T}}$. This closeness is the degree to which *T* accounts for *C*:

$$\begin{aligned} A(C,T):=G\Bigg (\frac{D_{0,0.3,1,1}\left( C, {\overline{T}}\right) }{N}\Bigg ), \end{aligned}$$

where *D* is a weighted Hamming Distance between arbitrary positions *P*, *Q*,

$$\begin{aligned} D_{d_0,d_1,d_2,d_3}\left( P,Q\right) := \sum _{\{s,\lnot s\} \subset S} d_{d_0,d_1,d_2,d_3}\left( P,Q,\{s,\lnot s\}\right) \; \end{aligned}$$

with the penalty function *d*:

$$\begin{aligned} d_{d_0,d_1,d_2,d_3}(P,Q,\{s,\lnot s\}) = {\left\{ \begin{array}{ll} d_3 \quad &{} \textrm{if} \quad \{s,\lnot s\} \subset (P\cup Q) \quad \\ d_2 \quad &{} \textrm{if} \quad \{s,\lnot s\} \cap P \ne \emptyset \wedge \{s,\lnot s\} \cap Q = \emptyset \quad \\ d_1 \quad &{} \textrm{if} \quad \{s,\lnot s\} \cap P = \emptyset \wedge \{s,\lnot s\} \cap Q \ne \emptyset \quad \\ d_0 \quad &{} \mathrm{otherwise.} \end{array}\right. }. \end{aligned}$$

The monotonically decreasing function *G* is chosen as

$$\begin{aligned} G(x):=1-x^2. \end{aligned}$$

Faithfulness is supposed to be a tie to the initial commitments. Its functional representation measures how close the commitments are to the initial commitments. The definition is almost completely analogous to the previous one. The only difference is that extending the initial commitments is not penalised:

$$\begin{aligned} F(C|C_0):=G\Bigg (\frac{D_{0,0,1,1}\left( C_0, C\right) }{N}\Bigg ). \end{aligned}$$

Lastly, a theory’s systematicity is a combination of its simplicity and scope. The number of the theory’s principles (minus 1) is divided by the number of sentences in its content. The result is plugged into the monotonically decreasing function *G*:

$$\begin{aligned} S(T) := G\Bigg (\frac{|T|-1}{|{{\overline{T}}} |} \Bigg ). \end{aligned}$$

As a result, *S*(*T*) increases with less principles and more content, as desired. The subtraction of 1 from the number of principles makes perfect systematicity $S(T)=1$ possible. Since this function is well-defined only for non-empty theories, $S(\emptyset ):=0$.

Now we trade off these desiderata to obtain an epistemic state’s achievement:

$$\begin{aligned} Z(C,T\vert C_0) := \alpha _F\cdot F(C\vert C_0) + \alpha _A\cdot A(T,C) + \alpha _S\cdot S(T), \end{aligned}$$

with non-negative weights $\alpha _F,\alpha _A,\alpha _S$ adding up to 1. As a standard configuration, $\alpha _F = 0.1,\ \alpha _A = 0.35,\ \alpha _S = 0.55$ has proven to provide plausible results. For more on this, see Beisbart et al.’s (2021) paper.

## Footnotes

1 In this paper, the abbreviation “TJ” refers to the 1999 revised edition.

2 In this paper, the abbreviation “PL” refers to the 2005 expanded edition.

## References

[CR1] Beisbart C, Betz G, Brun G (2021). Making reflective equilibrium precise: A formal model. Ergo.

[CR2] Betz, G. (2010). *Theorie dialektischer Strukturen*. Klostermann.

[CR3] Flick, S. (2022). *Ein realistischeres Modell des Überlegungsgleichgewichts*. Bachelor thesis, University of Bern.

[CR4] Freivogel, A., & Cacean, S. (2021). *Technical report: Assessing a formal model of reflective equilibrium*. University of Bern/KIT. https://www.philosophie.unibe.ch/unibe/portal/fak_historisch/dkk/philosophie/content/e40373/e82357/e776174/e1164904/e1365144/AssessingaFormalModelofReflectiveEquilibrium_ger.pdf

[CR5] Goodman N (1955). Fact, fiction, & forecast.

[CR6] Hegselmann R, Krause U (2002). Opinion dynamics and bounded confidence: Models, analysis and simulation. Journal of Artificial Societies and Social Simulation.

[CR7] Muldoon R, Borgida M, Cuffaro M (2012). The conditions of tolerance. Politics, Philosophy & Economics.

[CR8] Rawls, J. (1971). *A theory of justice*. Cambridge (Revised edition 1999).

[CR9] Rawls, J. (1993). *Political liberalism*. Columbia University Press (Expanded edition 2005).

